# Human CD34^+^ very small embryonic-like stem cells can give rise to endothelial colony-forming cells with a multistep differentiation strategy using UM171 and nicotinamide acid

**DOI:** 10.1038/s41375-022-01517-0

**Published:** 2022-02-15

**Authors:** Alison Domingues, Elisa Rossi, Kamila Bujko, Grégoire Detriche, Ulysse Richez, Adeline Blandinieres, Magdalena Kucia, Janina Ratajczak, David M. Smadja, Mariusz Z. Ratajczak

**Affiliations:** 1 Stem Cell Biology Program, University of Louisville, Louisville, KY 40245 USA; 2 Université de Paris, INSERM, Innovative Therapies in Haemostasis, F-75006 Paris, France; 3 Laboratory of Regenerative Medicine, Medical University of Warsaw, Warsaw, Poland; 4 Vascular Medicine Department and Biosurgical Research Lab (Carpentier Foundation), AP-HP, Hôpital Européen Georges Pompidou, F-75015 Paris, France; 5 Hematology Department and Biosurgical Research Lab (Carpentier Foundation), AP-HP, Hôpital Européen Georges Pompidou, F-75015 Paris, France

**Keywords:** Cell signalling, Medical research

## To the Editor:

Endothelial colony-forming cells (ECFCs) are now the consensus endothelial progenitor cells subtype with vasculogenic potential [1]. Ontogeny of ECFCs are still a matter of debate. Human very small embryonic-like stem cells (VSELs) are small dormant stem cells with properties of self-renewal and multipotential ability to differentiate in the three-germ layers [2]. We previously described that human VSELs from bone marrow are able to give rise to vessel formation in vivo [3] and several independent groups confirmed our data with human, mouse or rat VSELs [4]. Thus, we believe that ECFCs could originate from VSELs. To develop a source of ECFC-like cells for vascular repair, the use of human induced pluripotent stem cells (iPS) and embryonic stem cells (ESCs) has been investigated [5]. However, some significant molecular and functional differences have been observed between iPS-derived ESCs and primary ESCs. Beside low expression of NOS3 and retention of Oct4 and Klf4 expression in iPS-ESCs, these cells do not cluster properly with primary ESCs and posses immature mitochondria [5].

Therefore, VSELs could represent a clinically relevant alternative to iPS or ESCs, since they do not complete blastocyst development and do not form teratomas after transplantation into deficient mice [2]. Thus, in vitro expansion and differentiation of VSELs into ECFCs would represent a valuable source of ECFC-like cells for cell therapy and tissue engineering applications. The aim of our study is to propose a protocol using a chemically defined method to differentiate VSELs into ECFCs and open new perspectives for cell therapy and deciphering ECFCs ontogeny.

**Fig. 1:**
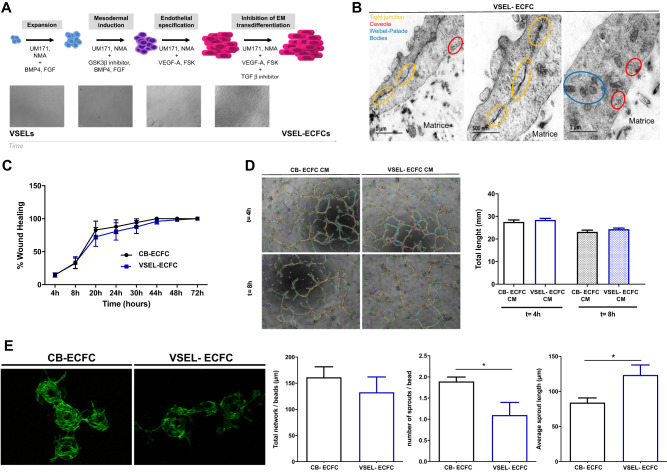
Sorted differentiated VSELs into ECs are similar to ECFCs. **A** Schematic illustration of the endothelial cell differentiation strategy from VSELs. The different steps over time are summarized and illustrated by phase images of the cells in these different steps along time. First, the VSEL phenotype was confirmed with the small-round-sized cells. After mesodermal induction and endothelial differentiation, cells are bigger and elongated. **B** Transmission electron microscopy images of VSEL-ECFC cultivated for 24 h on fibrin network on top of pericardial membrane: left panel represents large picture of VSEL-ECFCs on top of fibrin matrices, middle panel represents a higher magnification of the same region than left panel and right panel represent a region with potential Weibel-palade bodies and caveolae. **C** Longitudinal quantification of migrated VSEL-ECFCs and CB-ECFCs after scratch assay, expressed as a percentage of wound healing (*n* = 5 per group). Results are expressed as means ± SEM and were analyzed by two-way ANOVA, followed by a Bonferroni post hoc test. **D** Left panel: representative phase images of formation of pseudo-tube in Matrigel model in vitro after 4 and 8 h from ECFCs in conditioned medium from VSEL-ECFCs and CB-ECFCs. Right panel: quantification of formation of pseudo-tube, expressed as total length of tubes (*n* = 6 per group). Results are expressed as means ± SEM and were analyzed by two-way ANOVA, followed by a Bonferroni post hoc test. **E** In vitro and In vivo angiogenic properties of VSEL-ECFCs. Representative fluorescence images of the 3D sprouting assay. Quantification of the sprouting, expressed as total network per bead and, number of sprouts per bead and average sprout length (*n* = 6 per group). Results are expressed as means ± SEM and were analyzed using Wilcoxon test. *p ≤ 0.05.

We report here for the first time a multistep differentiation strategy of highly purified umbilical cord blood-derived VSELs based on CD34^+^ as previously described [6]. Culture of CD34^+^-VSELs is then realized including a mesodermal induction followed by endothelial specification (Fig. 1A). VSELs were identified and purified by prospective cell sorting as cells of appropriate size with a lineage-negative, CD45^−^, CD34^+^ phenotype (Supplementary Fig. 1A). Sorted VSELs were cultured in EGM-2 (Lonza) supplemented with 20% FBS on fibronectin coated plates at an average density of 50000 cells/cm^2^ in presence of stem cell culture adjuvants known to induce expansion, reprogramming and differentiation: UM-171 and nicotinamide acid (NMA). Until day 3, cells were cultivated and expanded in EGM-2 (Lonza) supplemented with 20% FBS with additional BMP-4 (5 ng/ml), FGF-2 (10 ng/ml), UM-171 (50 nM) and NMA (2.5 μM). Subsequently, cells were cultured in mesodermal differentiation medium, which is composed by the same cocktail with GSK3β inhibitor in addition for three over days. At day 6, the culture medium was switched to endothelial differentiation medium with EGM-2 (Lonza) supplemented with 20% FBS and in the presence of additional VEGF-A (300 ng/ml), FSK (2 μM), UM-171 (50 nM) and NMA (2.5 μM) until reaching confluence. During this step, sorted VSELs enlarged and displayed extended morphology with appearance of cobblestone cells (Fig. 1A). Endothelial cell commitment and differentiation were maintained by inhibiting potential mesodermal transition using TGF-β inhibitor after the first passage and until confluence is reached. After 16 ± 4 days of culture with this multistep differentiation protocol, we obtained a great number of cells (around 1.5 × 10^6^ differentiated cells when starting from ~16000 sorted CD34^+^ cells with characteristic cobblestone morphology of endothelial cells. Since VSELs-derived endothelial cells displayed morphological and phenotype similar to ECFCs isolated from the same cord blood (CB-ECFCs), we choose to name these cells as VSEL-ECFCs. VSEL-ECFCs were characterized by tight junctions, caveolae and Weibel-Palade Bodies (Fig. 1B), as demonstrated by transmission electron microscopy analysis of cell cultures conducted on fibrin network. Besides, VSEL-ECFCs presented the same levels of expression of endothelial markers when compared to CB-ECFCs (Supplementary Fig. 1B). To ensure the absence of mesenchymal phenotype acquisition we also analyzed the expression of Thy-1, a mesodermal marker. VSEL-ECFCs, as well as CB-ECFCs, were negative for Thy-1, confirming the endothelial phenotype of the expanded cells without acquisition of a mesenchymal phenotype (Supplementary Fig. 1B). We then explored functional and angiogenic properties of VSELs-ECFCs in contrast to CB-ECFCs. Migration properties evaluated with wound healing assay (Fig. 1C) and Boyden modified chamber (Transwell® assay, Supplementary Fig. 1C) demonstrated same ability of both cell type to migrate. However, VSEL-ECFCs showed significantly lower proliferative potential when compared to CB-ECFCs properties between CB- and VSELs-ECFCs (Supplementary Fig. 1D). We then explored angiogenic potential of conditioned medium by assessing growth factor secretion profile and pseudotubes formation in growth factor reduced Matrigel of control ECFCs. Cytokine profile (Supplementary Fig. 1E) as well as pseudotubes formation ability were both comparable between VSELs-ECFCs and CB-ECFCs (Fig. 1D). Moreover, vasculogenic potential of VSEL-ECFCs were explored using a 3D sprouting assay. VSEL-ECFCs were able to form sprouts but their ability seems lower than CB-ECFCs (Fig. 1E). Finally, in vivo angiogenic properties were evaluated by in vivo Matrigel implant assay. VSEL-ECFCs or CB-ECFCs contained in Matrigel (1.5 × 10^6^ cells (ECFCs or VSEL-ECFCs) 1.5 × 10^6^ MSCs + /200 µl of Matrigel/animal) were implanted subcutaneously in 8-week-old mice. Fourteen days after implantation, hematoxylin and eosin staining of Matrigel sections showed numerous vascular channels filled with red blood cells in the two different conditions (Supplementary Fig. 1E). Even if the diameter of the formed vessels is comparable in implants containing VSELs-ECFCs and CB-ECFCs, vessels number is increased in implants containing CB-ECFCs in contrast to VSELs-ECFCs.

In regenerative medicine, there is a significant effort for developing therapies based on endothelial progenitor cells such as ECFCs but their ontogeny is still unclear. VSELs are the most primitive stem cells in bone marrow, which can give rise to different tissue committed progenitor cells from all germ layers without confounding factor of feeder cell fusion with VSELs [2]. Endothelial cells derived from many cell sources through numerous protocols can be potentially delivered for regenerative medicine in patients with cardiovascular disease. In the literature, there are several papers with production protocols of ECFCs using hESCs or hiPSCs [5, 7**–**9]. To our knowledge, we are the first to propose a new valuable source of endothelial cells similar to ECFCs from characterized human stem cells that are not hESCs, hiPSCs or hemangioma stem cells. We described here a multistep differentiation culture recapitulating the developmental processes occurring during embryogenesis that allowed us to differentiate sorted CD34^+^-VSELs in ECFC-like cells. We then characterized VSELs-ECFCs angiogenic properties in vitro and vivo. First, VSELs, are known to be quiescent cells [10]. Thus, to overcome this status, we expanded sorted VSELs in presence of NMA and UM-171 compound. In fact, NMA is able to promote cell survival, cell differentiation and increased transcriptionally active chromatin [11]. UM-171 promotes cell self-renewal and improves cell expansion and proliferation [6]. We used FGF-2, VEGF-A and BMP-4 as it has been shown to synergistically induced early vascular progenitors from iPSC-derived mesodermal cells [7]. After a short expansion, VSELs are cultured in a chemically defined medium supplemented with a GSK3β inhibitor and BMP-4 inducing commitment of the cell population to the mesodermal state [7]. Cells are then cultured in a different medium containing VEGF-A and VSEL-ECFCs can be further expanded. Our protocol does not require embryoïd body formation or co-culture with other stromal cells/feeders. Endothelial cell commitment and differentiation through activation of VEGF-A signaling may be enhanced by the use of forskolin as suggested elsewhere [8, 12, 13]. Then, to maintain VSELs-derived endothelial cells we decided to prevent hypothetic endothelial-mesenchymal transdifferentiation by a TGF-β inhibitor treatment [14, 15]. We obtained promising results on the function and properties of ECFC-like cells derived from VSELs. Vasculogenic properties of VSELs have already been shown but with the differentiation cocktail used in this work we obtained cells with endothelial phenotype and characteristics, similar to ECFCs. We also demonstrated angiogenic ability of VSEL-ECFCs to form sprouts in vitro or vessels in vivo, even if this angiogenic potential is significantly decreased in contrast to CB-ECFCs. Their lower angiogenic properties could be due to a limited proliferation capacity. We were able to reverse VSELs-quiescent state and to differentiate them obtaining a large number of cells after few passages thanks to the use of UM-171 compound and NMA [6, 11]. Hence, further work is required to stimulate the proliferative capacity of VSELs without affecting their potential for differentiation as well as to elucidate the exact molecular mechanism controlling their endothelial differentiation.

All in all, we demonstrate for the first time here a full in vitro differentiation of VSELs in true vasculogenic progenitor ECFCs with a chemically identified media with UM-171 and NMA added to culture from the first day of culture. VSEL-ECFCs have the same endothelial morphology, phenotype and secretory potential than CB-ECFCs and have the ability to form functional vessels in a Matrigel implant model. Our study provides new insights in favor of ECFCs ontogeny and confirming interest of VSELs in vascular regeneration. Further studies need to better decipher origin of ECFCs and improve the vascular regenerative potential of VSELs by enhancing proliferative and vasculogenic potential of VSEL-ECFCs in vitro and in vivo and obtain highly efficient production of patient-derived VSEL-ECFCs for the treatment of vascular disease

### Supplementary information

Legend for Supplementary Figure 1

Supplementary Figure 1
