# Correction: Human CD34^+^ very small embryonic-like stem cells can give rise to endothelial colony-forming cells with a multistep differentiation strategy using UM171 and nicotinamide acid

**DOI:** 10.1038/s41375-023-01944-7

**Published:** 2023-09-18

**Authors:** Alison Domingues, Elisa Rossi, Kamila Bujko, Grégoire Detriche, Ulysse Richez, Adeline Blandinieres, Magdalena Kucia, Janina Ratajczak, David M. Smadja, Mariusz Z. Ratajczak

**Affiliations:** 1 Stem Cell Biology Program, University of Louisville, Louisville, KY 40245 USA; 2 Université de Paris, INSERM, Innovative Therapies in Haemostasis, F-75006 Paris, France; 3 Laboratory of Regenerative Medicine, Medical University of Warsaw, Warsaw, Poland; 4 Vascular Medicine Department and Biosurgical Research Lab (Carpentier Foundation), AP-HP, Hôpital Européen Georges Pompidou, F-75015 Paris, France; 5 Hematology Department and Biosurgical Research Lab (Carpentier Foundation), AP-HP, Hôpital Européen Georges Pompidou, F-75015 Paris, France

**Keywords:** Cell signalling, Medical research

Correction to: *Leukemia* 10.1038/s41375-022-01517-0, published online 15 February 2022

Owing to errors introduced during the proofreading process, the label « CB-ECFC » was erroneously added to the Figure. 1B and in the legend for panel 1B for the same Fig. 1. This panel shows VSELS-ECFC under different magnification. The original article has been corrected.

**Fig. 1:**
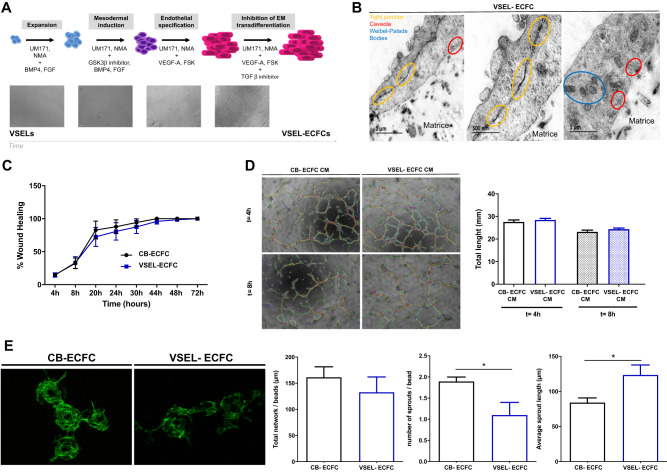
Sorted differentiated VSELs into ECs are similar to ECFCs. **A** Schematic illustration of the endothelial cell differentiation strategy from VSELs. The different steps over time are summarized and illustrated by phase images of the cells in these different steps along time. First, the VSEL phenotype was confirmed with the small-round-sized cells. After mesodermal induction and endothelial differentiation, cells are bigger and elongated. **B** Transmission electron microscopy images of VSEL-ECFC cultivated for 24 hours on a human fibrin network : left panel represent large picture of VSEL-ECFCs on top of fibrin matrice, middle panel represent a higher magnification of the same region than left panel and right panel represent a region with potential Weibel-palade bodies and caveolae. **C** Longitudinal quantification of migrated VSEL-ECFCs and CB-ECFCs after scratch assay, expressed as a percentage of wound healing (*n* = 5 per group). Results are expressed as means ± SEM and were analyzed by two-way ANOVA, followed by a Bonferroni post hoc test. **D** Left panel—Representative phase images of formation of pseudo-tube in Matrigel model in vitro after 4 and 8 h from ECFCs in conditioned medium from VSEL-ECFCs and CB-ECFCs. Right panel—Quantification of formation of pseudo-tube, expressed as total length of tubes (*n* = 6 per group). Results are expressed as means ± SEM and were analyzed by two-way ANOVA, followed by a Bonferroni post hoc test. **E** In vitro and In vivo angiogenic properties of VSEL-ECFCs. Representative fluorescence images of the 3D sprouting assay. Quantification of the sprouting, expressed as total network per bead and, number of sprouts per bead and average sprout length (*n* = 6 per group). Results are expressed as means ± SEM and were analyzed using Wilcoxon test. *p ≤ 0.05.

