# Supplementary material for: Human CD34+ very small embryonic-like stem cells can give rise to endothelial colony-forming cells with a multistep differentiation strategy using UM171 and nicotinamide acid
Source: Leukemia. 2022 Feb 15;36(5):1440–3. doi: 10.1038/s41375-022-01517-0 (PMC9061289; doi:10.1038/s41375-022-01517-0)
Supplement: Supplementary file 1 — Legend for Supplementary Figure 1 [file 41375_2022_1517_MOESM1_ESM.docx]

**Legend for Supplementary Figure 1.**

**(A)** Gating strategy for sorting of human VSELs derived by FACS; VSELs were identified within mononuclear cells (MNCs) according to size criteria, lineage^-^CD45^–^ CD34^+^ phenotype by flow cytometry. Figures showed here are the result from one bone marrow sample and is representative of all samples. **(B)** Representative flow cytometry analysis of CB-ECFCs and VSEL-ECFCs for endothelial markers; CD31, CD144, KDR, CD105. **(C)** **Upper panel** - Representative fluorescence images of migrated VSEL-ECFCs and CB-ECFCs by chemotactic migration assay (cells stained with DAPI). **Down panel** - Quantification of migrated VSEL-ECFCs and CB-ECFCs by chemotactic migration assay, expressed as number of cells per mm^2^ (n=5 per group). Results are expressed as means ± SEM and were analyzed using Kruskal-Wallis and Dunn’s multiple comparison tests. **(D)** **Upper panel** - Representative phase images of same number of seeded VSEL-ECFCs compared to CB-ECFCs at the same time point after proliferation. **Down panel -** Quantification of the proliferation ability, expressed as number of cells (n=7 per group). Results are expressed as means ± SEM and were analyzed by 2-way ANOVA, followed by a Bonferroni post hoc test. *p≤0.05**. (E)** Heat map generated based on proteins expression levels for the analysis of pro-angiogenic factors secreted by VSEL-ECFCs and CB-ECFC. Heat-map indicates no differences in the levels of secreted pro-angiogenic factors (red = higher expression, blue = lower expression) (n=5 per group). **(F)** Representative phase images of H&E-stained Matrigel plug sections seeded with 1,5 x10^6^ VSEL-ECFCs and 1,5 x10^6^ MSCs or 1,5 x10^6^ CB-ECFCs and 1,5 x10^6^ MSCs, showing the formation of functional vessels (↓) and magnification. Quantification of *in vivo* vessel formation, expressed as number of vessels, and average diameter length (n=5 per group). Results are expressed as means ± SEM and were analyzed using Wilcoxon test. *p≤0.05.
