# Supplementary material for: Human CD34+ very small embryonic-like stem cells can give rise to endothelial colony-forming cells with a multistep differentiation strategy using UM171 and nicotinamide acid
Source: Leukemia. 2022 Feb 15;36(5):1440–3. doi: 10.1038/s41375-022-01517-0 (PMC9061289; doi:10.1038/s41375-022-01517-0)
Supplement: Supplementary file 2 — Supplementary Figure 1 [file 41375_2022_1517_MOESM2_ESM.pptx]

## Slide 1
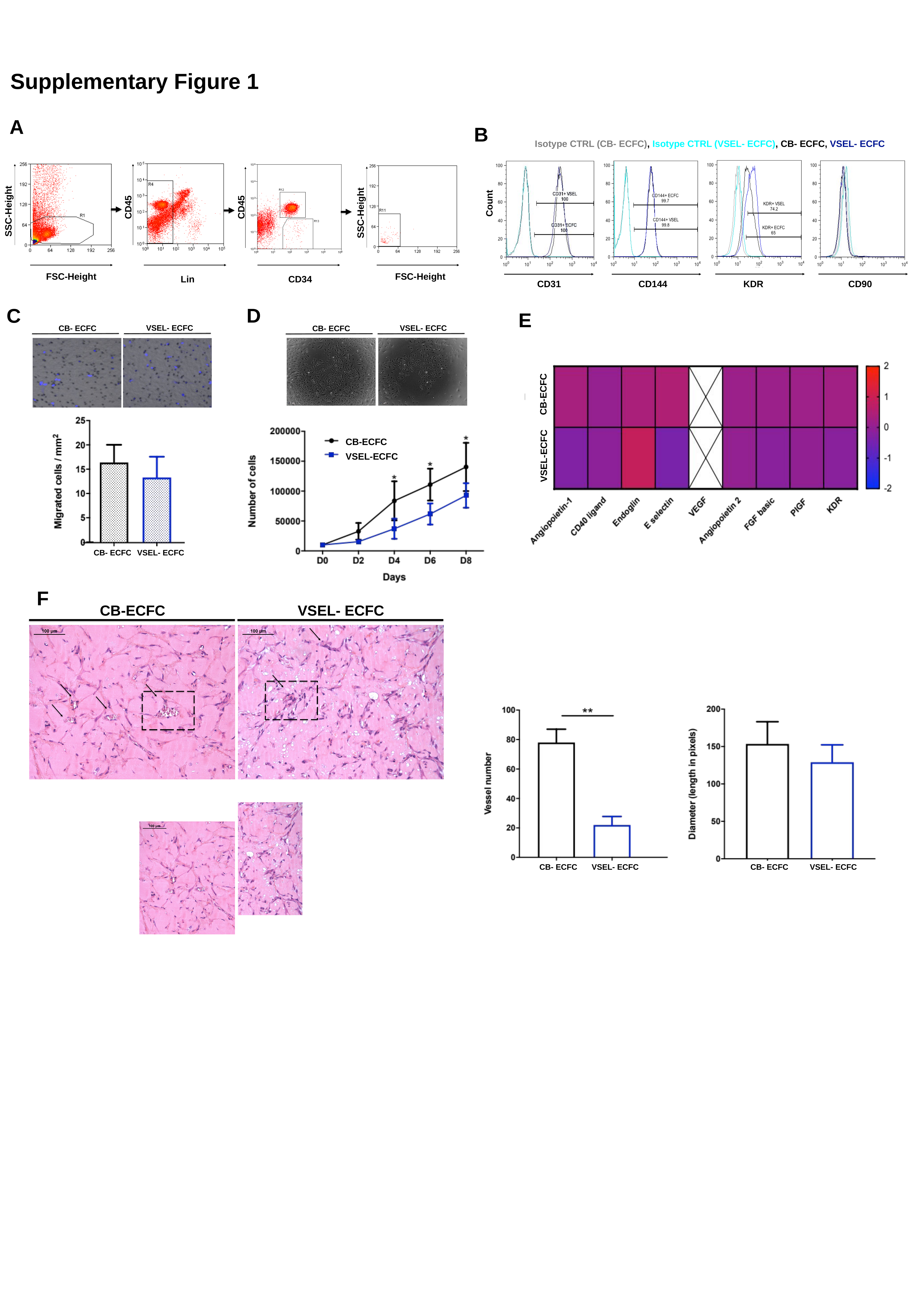

Supplementary Figure 1
A
B
Isotype CTRL (CB- ECFC), Isotype CTRL (VSEL- ECFC), CB- ECFC, VSEL- ECFC
Count
CD45
CD45
SSC-Height
SSC-Height
FSC-Height
FSC-Height
Lin
CD34
CD31
CD144
CD90
KDR
D
C
E
VSEL- ECFC
VSEL- ECFC
CB- ECFC
CB- ECFC
CB-ECFC
CB- ECFC
VSEL- ECFC
CB-ECFC
VSEL-ECFC
VSEL-ECFC
F
CB-ECFC
VSEL- ECFC
CB- ECFC
VSEL- ECFC
CB- ECFC
VSEL- ECFC
